# Polymeric Transducers: An Inkjet Printed B-Field Sensor with Resistive Readout Strategy

**DOI:** 10.3390/s19235318

**Published:** 2019-12-03

**Authors:** Bruno Andò, Salvatore Baglio, Ruben Crispino, Vincenzo Marletta

**Affiliations:** DIEEI, University of Catania, Viale A. Doria 6, 95125 Catania, Italy; bruno.ando@unict.it (B.A.); salvatore.baglio@unict.it (S.B.); vincenzo.marletta@diees.unict.it (V.M.)

**Keywords:** magnetic field sensor, printed sensor, magneto-mechanical interaction

## Abstract

Magnetic field sensors are successfully used in numerous application contexts such as position sensing, speed detection, current detection, contactless switches, vehicle detection, and electronic compasses. In this paper, an inkjet printed magnetic sensor, based on the magneto-mechanical sensing principle, is presented together with a physical model describing its physical behavior and experimental results. The main novelties of the proposed solution consist of its low cost, rapid prototyping (printing and drying time), disposability, and in the use of a commercial low-cost printer. A measurement survey has been carried out by investigating magnetic fields belonging to the range 0–27 mT and for different values of the excitation current forced in the actuation coil. Experimental results demonstrate the suitability of both the proposed sensing strategy and model developed. In particular, in the case of an excitation current of 100 mA, the device responsivity and resolution are 3700 µε/T and 0.458 mT, respectively.

## 1. Introduction

Inkjet printing technology allows to develop low-cost sensors and electronics into flexible substrates [1,2,3,4,5], both for mass production and rapid prototyping [6,7,8]. Main advantages of this technology reside in the maskless approach and the direct printing technique that allow to reduce the waste of ink and time to production [9].

The printing processing needs three main ingredients to be fixed: the kind of inkjet printer, conductive or functional inks, and the substrate.

In case the printing task is not really demanding in terms of resolution and variety of inks, customized inks can be printed by common office printers. Otherwise, in case high resolution and specific inks are required, really sophisticated printers are currently available on the market [10,11,12,13,14,15].

Typical conductive inks include metallic nanoparticles (NPs) and nanowires, and carbon-based materials, such as carbon nanotubes and graphene. Several alternative conductive inks can be found on the market, including organometallic and conductive polymeric inks. The choice of the most suitable material depends on the specific application requirements. NP inks usually consist of a suspension of gold or silver particles, in water or in an organic solvent, and are characterized by high chemical stability and high electrical conductivity [10]. The presence of water in the suspension requires the addition of an ionic surfactant to disperse the conductive materials and a heat treatment, known as sintering, to be carried out after the printing process. Water-based inks are safer and easier to handle than solvent-based inks, which can be corrosive and potentially harmful, but have faster drying times than water-based inks. Organometallic inks are solutions of metal compounds dissolved in organic solvents. The risk of clogging the printer head nozzles is strongly reduced because they are in the form of solutions rather than particle suspension. Furthermore, higher conductivity values can be achieved and lower sintering temperatures are required compared to NP inks. Although silver-based organometallic inks are commonly used, there is a growing interest in graphene-based inks [10,11,12]. Among polymeric inks, PEDOT: PSS and Polianilina (PANI) [13] are commonly used being compatible with inkjet printing systems. The combination of conductive and functional inks has been proposed as a cost-effective solution for producing sensors by means of inkjet printing technology [10].

Metallic inks in combination with low-cost printers have been used successfully for rapid prototyping of sensors [6,15]. Depending on the used printing equipment, inkjet printing can be used on rigid substrates (plastic, glass, ceramics, and silicon) or flexible (polyether imide, polycarbonate, polyacrylate, polyamide, polyethylene, terephthalate, and even paper) [10,16,17].

In the following, the development of an inkjet magnetic field sensor carried out at the SensorLab of the University on Catania, Italy, based on the magneto-mechanic principle, is presented.

## 2. The Inkjet Printed (IJP) B-Field Sensor

Magnetic field sensors are used in numerous application contexts such as position sensing, speed detection, current detection, contactless switches, vehicle detection, and electronic compasses, just to cite a few [18,19,20,21,22,23].

In case of environments classifiable as hostile due to aggressive or contaminating agents, it is important to have low-cost and intrinsically disposable sensors. Various sensing strategies for the measurement of magnetic field are reported in the literature, such as the magneto-mechanical transduction, the magnetic induction, the Hall effect, the magneto-resistive effect, and also the superconducting quantum interference devices, also known as SQUID [24].

The magneto-mechanical transduction, which consists in converting the action of a magnetic field into a mechanical deformation subsequently converted into an electric signal variation, represents one of the most economical and simplest solutions. For this reason, this method has been widely used, as an example, in different sensory solutions realized in MEMS technology or in realizing many types of relay. 

As an example, in [25] is presented a MEMS resonant magnetic sensor whose resonant vibration is excited by the Lorentz force. The sensor, meant to be integrable in an inertial measurement unit (IMU), has been fully designed, simulated, and realized. Results show that, with a current of 5 mA, the sensitivity of the sensor is 43 aF/mT.

Sonmezoglu et al. [26] have developed a dual-resonator MEMS magnetic sensor using the Lorentz force. The sensor readout strategy is based on frequency measurements, where the magnetic field strength is computed by monitoring the change in oscillation frequency. Being a differential structure, the two oscillators provide built-in temperature compensation. Measurement results demonstrated that by using a 1 mA bias current, the device has a sensitivity of 2180 Hz/T.

Another interesting work is the one presented in [27]. The authors describe a magnetic sensor, always based on the detection of Lorentz force on a micromechanical oscillator, whose operation is demonstrated using an amplitude modulation (AM) and a frequency modulation (FM) readout of the magnetic signal. The sensitivity of the sensor is 500 Hz/T with a noise floor of 500 nT/Hz^1/2^. Results show that the FM readout strategy performs better than the AM one since it allows a much greater bandwidth.

In [28] a MEMS magnetic field sensor with a capacitive readout strategy is presented. The sensor only detects the magnetic field in the orthogonal direction to the resonance structure surface. It consists of a set of fixed stators and a shuttle suspended with two thin beams that form two differential parallel-plate sensing capacitors, C1 and C2. A Lorentz force, generated on the two thin beams, is in the orthogonal direction to the plane of both magnetic field and ac current, causing a displacement of the beams and parallel plates. This displacement is detected through the differential capacitance variation between the parallel plates and fixed stators. The sensor sensitivity, measured as the differential capacitance shift per variation of magnetic field, is 150 µV/µT at 250 µA of peak driving current with a resolution of 520 nT/mA·Hz^1/2^.

A magnetic sensor’s readout strategy is based on the piezoresistors resistance variation is given in [29]. This sensor is made up by a rectangular loop of silicon beams, an aluminum loop, and a Wheatstone bridge. The sensor structure has a deflection and strain generated by the Lorentz force, causing a change of the initial resistance of two p-type piezoresistors. It has a resonant frequency of 22.99 kHz, a sensitivity of 1.94 V/T with a resolution close to 43 nT for a frequency difference of 1 Hz.

The possibility of combining the aforementioned sensing principle with a technology compliant with flexible substrates, which undergoes bending under the action of the target magnetic field, is a very interesting solution for the rapid prototyping of low-cost sensors.

In this paper, an inkjet printed magnetic field sensor, based on the magneto-mechanical sensing principle, is presented along with the developed mathematical model describing its physical behavior.

A preliminary investigation on this type of sensor is given in [30,31] where both the electrical and physical characteristics have been determined. Nevertheless, main novelties compared to the cited work are described in the following: (1) Description of the sensor mathematical model; (2) Computation of the sensor responsivity; (3) Model results compensation for the effect of tolerances in the device geometry and technology.

Main advantages of the proposed solution consist of its low cost, its rapid realization (printing and drying time), and disposability. 

**Figure 1:**
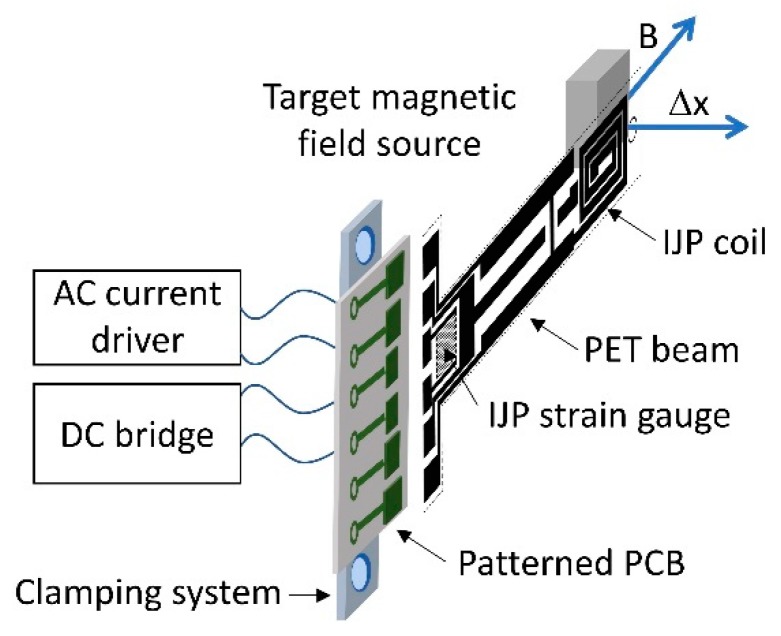
Schematization of the sensing principle.

The sensing principle of the device developed, schematized in Figure 1, is based on the well-known Lorentz force. The interaction between the unknown magnetic field and an alternating driving current flowing on a coil printed on the polyethylene terephthalate (PET) substrate produces a beam deflection, Δx, which can be converted into an electrical output by means of a resistive sensing strategy, implemented by means of a IJP strain gauge, and a dedicated conditioning electronics. 

### 2.1. The Physical Model

In order to correctly design the printed elements of the sensor, based both on the measurement specifications and constraints given by the specific applications, a mathematical model, describing the relationship between the unknown magnetic field and the generated strain, is proposed.

The developed model is introduced below while, a legend introducing terms used for the equations, is given in Table 1.

As first, it is necessary to fix constraints coming from the specific application and the technology. In particular, these are represented by the size of the beam (*W_beam_*) and the technology dependent parameters (*D_in_*, *D_guard_*, *S*), respectively. In particular, *D_in_* is considered a technology depended parameter since it is the dimension required by the pith used to close the loop. The model descriptions is aimed at the estimation of the optimal geometry of the inkjet printed actuation coil.

**Figure 2:**
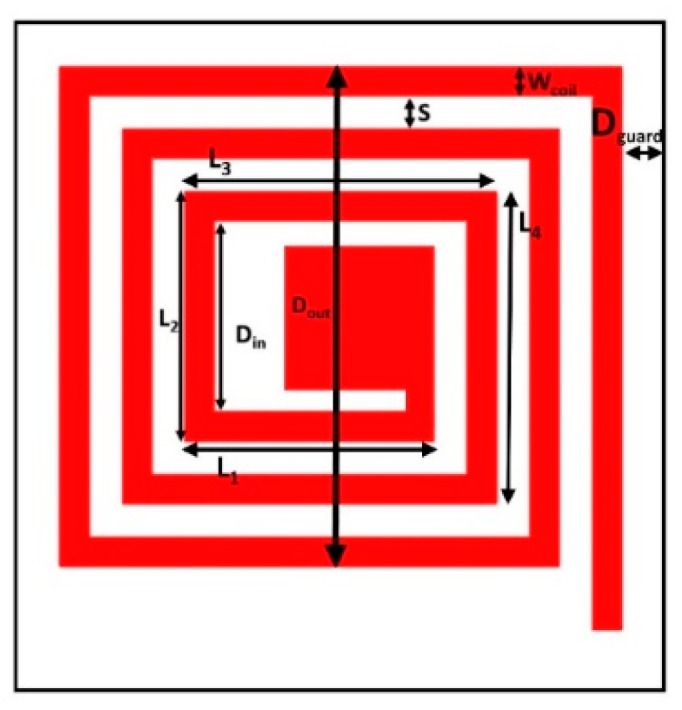
Layout of a squared coil.

With reference to Figure 2, which shows a squared quoted coil, the following relationships can be determined:

(1) $$D_{out}=W_{beam}\;-2D_{Guard}$$

(2) $$D_{out}=D_{in}+2W_{coil}+2\left(W_{coil}+S\right)\left(N-1\right)$$

Equation (2) can be used to determine the coil external diameter. Equation (2) allows us to estimate the number of nominal turns, *N_nom_*:

(3) $$N_{nom}=\frac{D_{out}-D_{in}-2W_{coil}}{2W_{coil}+2S}+1$$

Since, for the sake of device realization (Figure 1), only integer values of *N* can be considered, the coils number is rounded to the closest integer number:

(4) $$N=int(\frac{D_{out}-D_{in}-2W_{coil}}{2W_{coil}+2S}+1)$$

This procedure allows us to find the eligible couples (*N*, *W_coil_*) for a specific beam geometry.

Since the working principle is based on the interaction between the target magnetic field $\vec{B}$ and the current flowing on the coil *I*, the highest current density supported by the beam for a given couple (*N*, *W_coil_*) must be addressed.

The current density is limited by the maximum thermal power dispersible by the beam substrate, in order to avoid a permanent deformation of the beam material.

This value has been experimentally determined by using a coil sample printed on a PET beam, with *W_coil_* equal to 2.0 mm and *T_c_* equal to 200 nm (constrained by the printing technology). The shape of the beam has been observed for increasing values of the current forced into the coil. Several repetitions of this experiment have been accomplished, leading to the estimation of a maximum current, which does not produce any kind of deformation of the beam, equal to 130 mA. Exceeding this value will cause the permanent deformation of the beam substrate.

Hence, the maximum current density, *D_current_*, can be computed as follow:

(5) $$D_{current}=\frac{130\;\mathrm{mA}}{200\;\mathrm{nm}\cdot 2\;\mathrm{mm}}$$

Finally, a generalized relation can be determined for the maximum current supported by the coil/beam:

(6) $$I_{max}=W_{coil}\cdot T_{c}\cdot D_{current}$$

Since, as it will be discussed in Section 2.2, the realized sensor shares the same geometrical parameters of the sample used for the current density determination, its maximum current value is also 130 mA. Keeping this into account, a safety margin around 20% has been chosen, which leads to a maximum current value of 100 mA to be used during the experiments.

**Figure 3:**
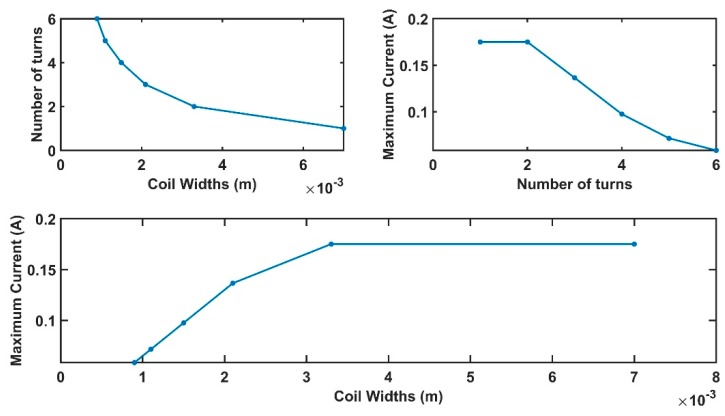
Results regarding the relation between *N*, *W_coil_*, and the maximum current, *I_max_*, supported by the beam. The currents addressed belong to the range 0–170 mA.

Results for the above equations, in the case of *W_coil_* belonging to the range of 0.9–7 mm, changing with a step of 0.1 mm, and *W_beam_* equal to 2 cm, are shown in Figure 3.

As it will be discussed in the next paragraphs, achieved results must be matched with the predicted behavior of the beam strain (ε) as a function of the target magnetic field ($\vec{B}$).

The beam strain due to an external magnetic field is given by [32,33]:

(7) $$\varepsilon =\frac{6\cdot F_{m}\cdot L_{Beam}}{E\cdot W_{beam}\cdot T_{Beam}^{2}}$$

where *F_m_* is the Lorentz force between the target magnetic field ($\vec{B}$) and the current flowing on the coil.

Assuming the orthogonality between the target magnetic field and the current *I*, the following relationship can be assumed:

(8) $$F_{m}=I\cdot P\cdot \vec{B}$$

where *P* is the coil perimeter.

The dependence on the conductor orientation has been omitted since we assume its effect is negligible at this modeling phase for the overall sensor model. This choice comes from previous results [30] where it has been demonstrated that the maximum beam deflection, and hence the maximum angle variation between current and target magnetic field, does not affect “very much” the model output (compared with the experimental measures). This point will be clarified in Section 3.

The side of a single loop turn, *L_i_*, with reference to Figure 2, can be obtained by the following relationships:

(9) $$L_{1}=L_{2}=2W_{coil}+D_{in}$$

(10) $$L_{3}=L_{2}\;+\;S\;+\;W_{coil}$$

By reiterating the process for the subsequent turns, we obtain:

(11) $$L_{i}=L_{i-2}+\left(S+W_{coil}\right)\;\mathrm{for}\;i=4,\;5,\;\ldots ,\;L_{\mathrm{tot}}$$

Starting from above relationships, *P* can be estimated as:

(12) $$P=\overset{L_{tot}}{\underset{i=1}{\sum }}L_{i}$$

**Figure 4:**
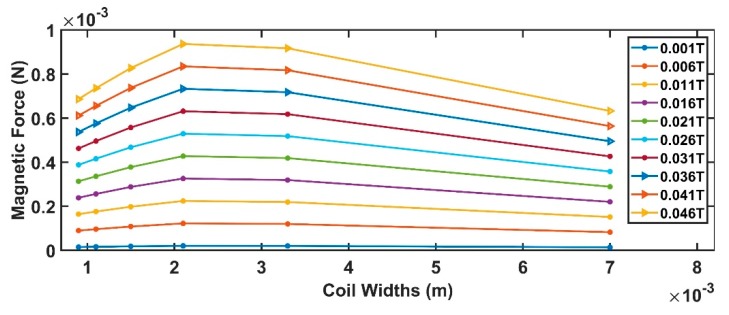
Relationship between the coil widths and the generated magnetic force.

**Figure 5:**
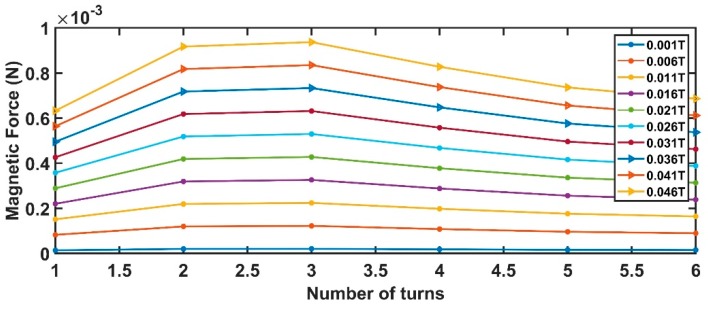
Relationship between the number of turns and the generated magnetic force.

**Figure 6:**
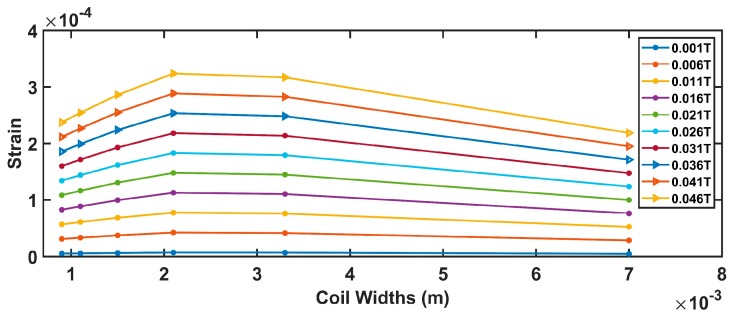
Relationship between the coil width and the strain.

**Figure 7:**
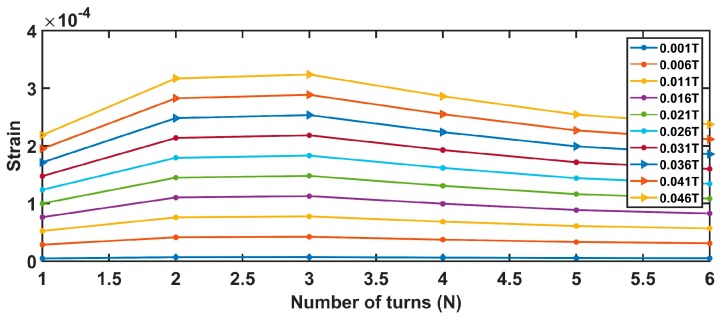
Relationship between the number of turns and the strain.

Assuming values for the device parameters given in Table 2, Equations (7)–(12) produce the results shown from Figure 4, Figure 5, Figure 6 and Figure 7.

Figure 4 contains the *W_coil_* value maximizing the sensor magnetic force (in the range of currents addressed by this work). If the application can sustain a greater current, compared to the one imposed in the present work, the pick can be obtained for larger *W_coil_* producing larger magnetic forces.

Figure 5 is strictly linked to the previous one. Since the number of coils and coil widths are in an inverse dependency, an increase in one produces the reduction of the other. Also, in this case, a pick is obtained for a number of turns equal to 3.

Comparable conclusions can be drawn from both Figure 6 and Figure 7. The strain is maximized for the values, also optimizing the magnetic force.

Finally, considering the comments above, the optimal system parameters assuring the maximization of the beam strain ε are *N =* 3 and *W_coil_* = 2 mm.

### 2.2. Device Realization and Experimental Setup

**Figure 8:**
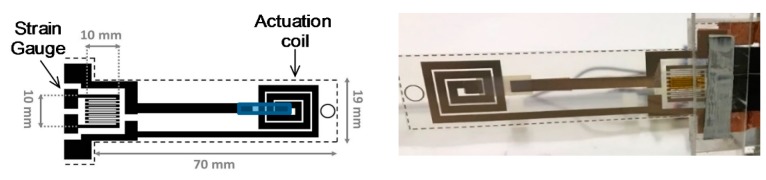
Schematization and a real view of the sensor. The device has a dimension of 70 mm by 19 mm, with a total thickness of 200 µm.

Following the parameters estimated in the previous section, a real device, whose schematization and a real view are shown in Figure 8, has been realized.

Specifically, the sensor consists of a PET beam fixed at one end, working also as the substrate for the realization of the actuation planar coil and the resistive readout strategy (strain gauge). A brief explanation of the working principle has already been given in Section 2 (for more details: [31]). Electrical characteristics of the printed devices are described in Table 3 [31].

The adopted printer is a commercial Deskjet, while, the silver-based conductive ink, is the Metalon^®^ JS-015 provided by NovacentrixTM.

## 3. Results

**Figure 9:**
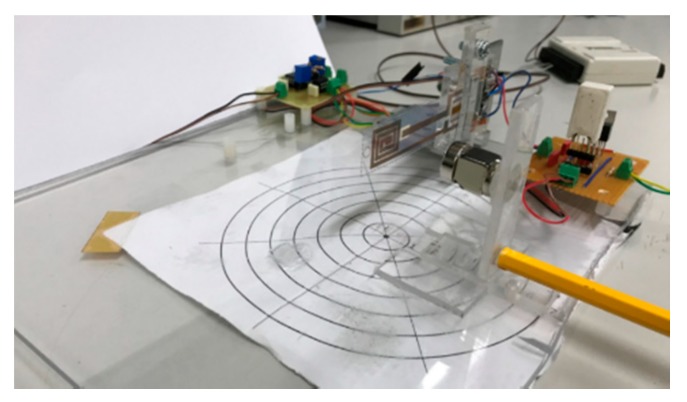
Experimental setup.

In order to assess the real behavior of the sensor the dedicated experimental setup, shown in Figure 9, has been used.

The target magnetic field is generated by a permanent magnet positioned at different distances from the IJP sensor.

**Figure 10:**
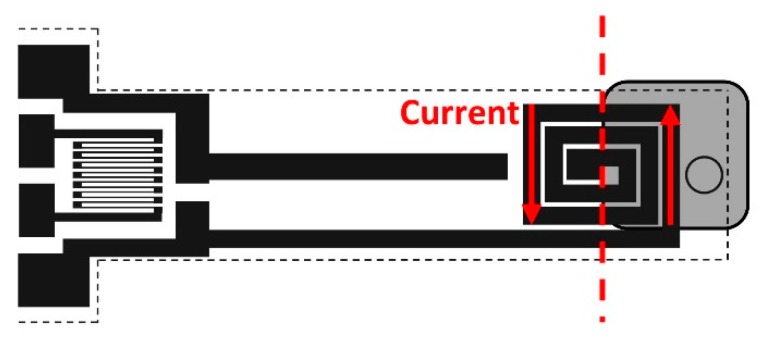
A detail of the magnet position compared to the coil and excitation current plane.

The setup aim is two-fold: to find the relationship between the permanent magnet distance and the magnetic field measured at the sensor location; measuring the device strain as a function of the magnetic field strength. The strength of the target magnetic field as a function of the distance has been characterized by means of a Hall effect sensor (SS496A1 by Honeywell). The target magnetic field, applied orthogonally with respect to the printed coil current plane (Figure 10), interact with a sinusoidal excitation current, whose frequency matches with the beam mechanical resonance frequency (9.1 Hz). This last choice allows us to maximize the device sensitivity. Having in mind the magnetic fluxes produced by a permanent magnet, just a part of it is useful to the sensor functionality: the ones parallel to the coil current plane. Although the magnetic stray field of the permanent magnet tends to quickly decay during the sensor oscillations, considering the small deflection of the sensor compared to the steady-state [30], its effect has not been fully investigated and hence, has been neglected in this modeling phase.

In order to clarify the experimental conditions, some additional considerations must be done. The first deals with the magnet position as respect to the printed coil. In order to have a positive net force applied to the beam, the magnetic flux must interact only with half of the coil (the part positioned on the tip of the sensor) and not in its entirety (as shown in Figure 10). This constraint is mandatory for the sensor to oscillate since the current flowing in the opposite coil direction will not produce any unwanted force. A second deals with the coil design. The coil layout has been optimized in such a way to enhance the effect of the Lorentz force in one direction: as it can be noticed in Figure 10, from right to left, the coil has an uneven number of coil segments along the beam width. The coil segments close to the tip are 3 while, in the other direction, 2 (the additional segment is a contact point which is used to place the conductive ribbon).

The measurement survey has been carried out by investigating magnetic fields belonging to the range 0–27 mT, by forcing the following excitation currents: 0.02 A, 0.04 A, 0.06 A, 0.08 A, and 0.1 A.

**Figure 11:**
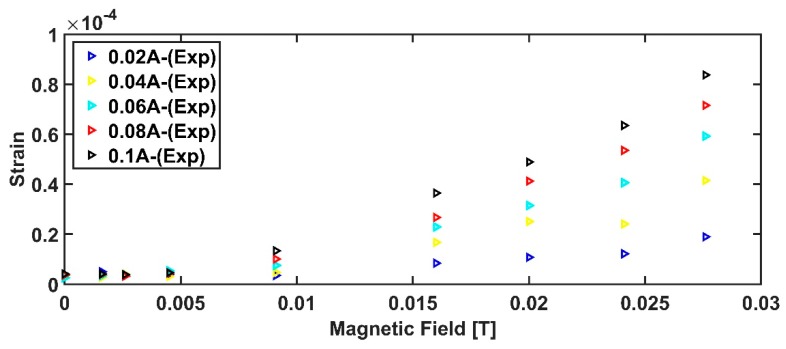
Sensor’s behavior as a function of the target magnetic field and the excitation current.

Results arising from the measurement surveys are shown in Figure 11. As it can be observed, in case of low excitation currents, the device shows a very poor responsivity, especially in the lower part of the working range. This is due to the need of overcoming the beam inertia. For this reason, the results coming from that combination have been removed both from the mathematical analysis and from the model fitting.

In order to compensate the model prediction, with respect to the real behavior, for the effect of tolerances in the device geometry and technology, the following relationship has been used:

(13) $$\varepsilon_{obs}=K_{1}\cdot \varepsilon +K_{2}$$

where the coefficients *K*_1_ and *K*_2_ are excitation current dependent. Their values can be found in Table 4.

The coefficient current dependency can be explained considering the simplifications adopted during the model development. In particular, the ones concerning the perfect orthogonality between the current and the target magnetic field. In reality, this angle depends on the coil excitation current (a greater excitation current produces an increased beam deflection). This variation, being a function of the current, produces compensation values dependent on the current itself.

Nevertheless, the small variation of K1 and K2 over the current intensity, further confirm the assumption that the deviation from the orthogonality is negligible.

**Figure 12:**
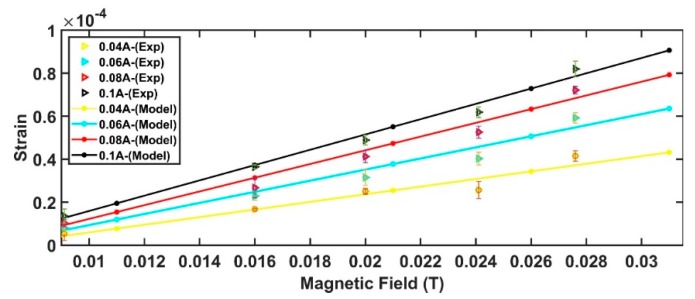
Model predictions and experimental data.

The expected behavior predicted by Equation (13) superimposed with the experimental data is shown in Figure 12.

Estimated values of the device responsivity, accuracy, and resolution are shown in Table 5. For the sake of completeness, the responsivity is defined as the slope of the output characteristic curve (Δy/Δx) shown in Figure 12, the accuracy as the standard deviation of the strain measurements as respect the model value while, the resolution, as the ratio between the strain’s standard deviation and responsivity.

As expected, by increasing the excitation current, a general improvement in the sensor performances can be observed.

These results confirm the validity of the developed model that can represent a powerful tool for the design of the inkjet printed magnetic field sensor.

## 4. Conclusions

In this paper, an inkjet printed magnetic sensor, based on the magneto-mechanical sensing principle, is presented. In particular, the device, fully inkjet printed with a commercial and low-cost printer, exploits the Lorenz force to actuate a flexible beam where a resistive readout strategy has been integrated. The main novelties of the proposed solution consist of its low cost, its fast prototyping, and disposability. These advantages make this specific kind of sensors suitable for applications requiring a disposable device, as well as for educational purpose and the realization of fast lab-scale prototype. Moreover, the use of a flexible substrate allows the device to be compliant with real scenario requiring flexible and shapeable sensors.

A mathematical model of the sensor has been proposed where both technology and application dependent parameters have been completely integrated. Two major advantages can be highlighted from the model: the possibility to predict the sensor’s behavior during design phases and to find the optimal physical parameters maximizing the sensor responsivity for the production phase.

A measurement survey has been carried out by investigating magnetic fields belonging to the range 0–27 mT and for the following excitation currents: 0.02 A, 0.04 A, 0.06 A, 0.08 A, and 0.1 A. Experimental results demonstrate the suitability of the proposed sensing strategy and the model developed.

Although the sensor responsivity has been limited by application constraints, there are quite a few options that can be adopted to increase it any further:

Geometry dependent:

- Increase the beam width *W_beam_* (this is strictly correlated to the application constraints).
- Increase the coil thickness *T_c_* (this is a matter of technology).
- Increase the beam length *L_beam_* (this is strictly correlated to the application constraints).

Electric quantity:

- Maximizing the coil excitation current, by taking into account the sensor geometry (this increase must always be compliant with the maximum current density “*D_current_*” compatible with the coil track geometry).

Although this paper addresses the device behavior in the case of the beam not pre-bended, future efforts will be dedicated to investigating such cases.

## Figures and Tables

**Table 1:** Physical and electrical quantities employed for the model description.

Physical Quantity	Value	Description
*N*		Number of coils turns
*W_coil_*		Coil width
*T_c_*	200 nm	Coil thickness (technology dependent)
*S*	300 µm	The lowest possible spatial resolution (technology dependent)
*D_in_*	2.0 mm	Internal coil Diameter (technology dependent)
*D_out_*		External coil Diameter (application dependent)
*D_guard_*	2.0 mm	External safety ring (technology dependent)
*L_i_*		Turn side
*L_tot_*		Total turn sides
*P*		Total coil length (taken considering the external coil diameter)
*W_beam_*		Beam width (application dependent)
*µ*_0_	4π·10^−7^ H/m	Magnetic permeability in vacuum
*I*		Current flowing in the beam
*I_max_*	130 mA	The highest current supported by the beam (application and technology dependent)
*D_current_*		Maximum current density
$\vec{B}$		External magnetic field
*E*	3.1·10^9^ N/m^2^	PET Young’s modulus
*L_beam_*		Beam length
*T_beam_*		Beam thickness
*F_m_*		Lorentz force

**Table 2:** Device parameters adopted to simulate the system behavior.

Physical Quantity	Value
*W_beam_*	2 cm
*T_c_*	200 nm
*S*	300 µm
*D_in_*	2 mm
*D_out_*	2 cm
*B*	1–46 mT with 5 mT step
*W_coil_*	0.9–7 mm with 0.1 mm step

**Table 3:** Electrical characteristics of the device.

Physical Quantity	Value
Coil resistance	25.6 Ω
Coil inductance	84.6 nH
Strain gauge resistance	123.5 Ω
Gauge factor	1.9
Resonant frequency	9.1 Hz

**Table 4:** Fitting constant for the strain computation.

Constant	0.04 A	0.06 A	0.08 A	0.1 A	Mean Value	STD
*K*_1_	0.910	0.883	0.818	0.730	0.835	0.079
*K*_2_	−0.117 × 10^−4^	−0.164 × 10^−4^	−0.196 × 10^−4^	−0.196 × 10^−4^	−0.169 × 10^−4^	3.73 × 10^−6^

**Table 5:** Responsivity, accuracy, and resolution of the sensor as a function of the excitation current.

Quantity	0.04 A	0.06 A	0.08 A	0.1 A
Responsivity (µε/T)	1800	2500	3100	3700
Resolution (mT)	0.864	0.644	0.486	0.458
Accuracy (µε)	±3.45	±2.82	±1.90	±1.50
